# Supplementary material for: Early life stress disrupts intestinal homeostasis via NGF-TrkA signaling
Source: Nat Commun. 2019 Apr 15;10:1745. doi: 10.1038/s41467-019-09744-3 (PMC6465335; doi:10.1038/s41467-019-09744-3)
Supplement: Supplementary file 1 — Supplementary Information [file 41467_2019_9744_MOESM1_ESM.docx]

**Supplementary Information**

Early life stress disrupts the intestinal homeostasis via NGF-TrkA signaling

Wong et al.

**Supplementary Figure 1**

**
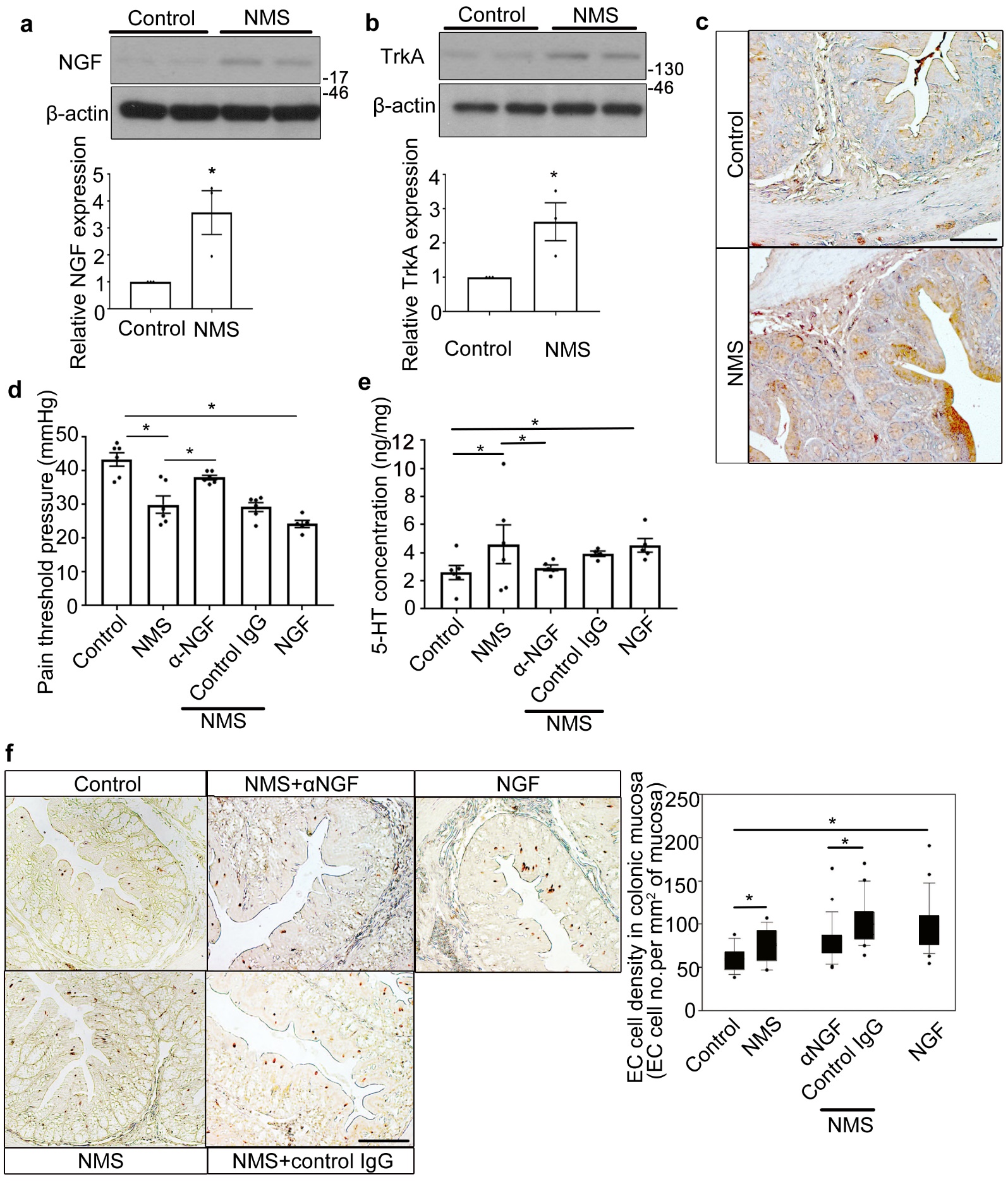
**

**Supplementary Figure 1. NMS leads to EC cell hyperplasia, increased enteric serotonin and visceral hyperalgesia through NGF/TrkA signaling**

**(a-b)** Western blotting analyses of NGF **(a)** and TrkA **(b)** expression in proximal colon tissues from NMS and control rats. Quantification of their expression levels was shown in the lower panel. (*p<0.05, n=3, two-tailed *t*-test). **(c)** Immunostaining using specific antibody against TrkA in proximal colon tissues from NMS and control rats**.** (scale bar: 100μm) **(d)** Assessment of visceral pain threshold in control and NMS rats treated with or without intraperitoneal injection of neutralizing antibodies against NGF (anti-NGF) and recombinant NGF protein. (*p<0.05, n≥5/group; ANOVA). **(e)** Concentration of serotonin (5-HT) was measured in the proximal colonic tissues from control and NMS rats treated with or without anti-NGF and NGF by means of capillary electrophoresis. (*p<0.05, n≥5/group; ANOVA). **(f)** Representative silver-staining images showing EC cells in different experimental groups from control and NMS rats treated with or without anti-NGF and NGF (left panel). (scale bar: 100μm) Quantification for EC cell density (the number of EC cells per mm^2^ of colonic mucosa) in different experimental groups was shown in the right panel. (*p<0.05, n≥5/group; ANOVA). All data represent the mean ± SEM**.**

**Supplementary Figure 2**

**
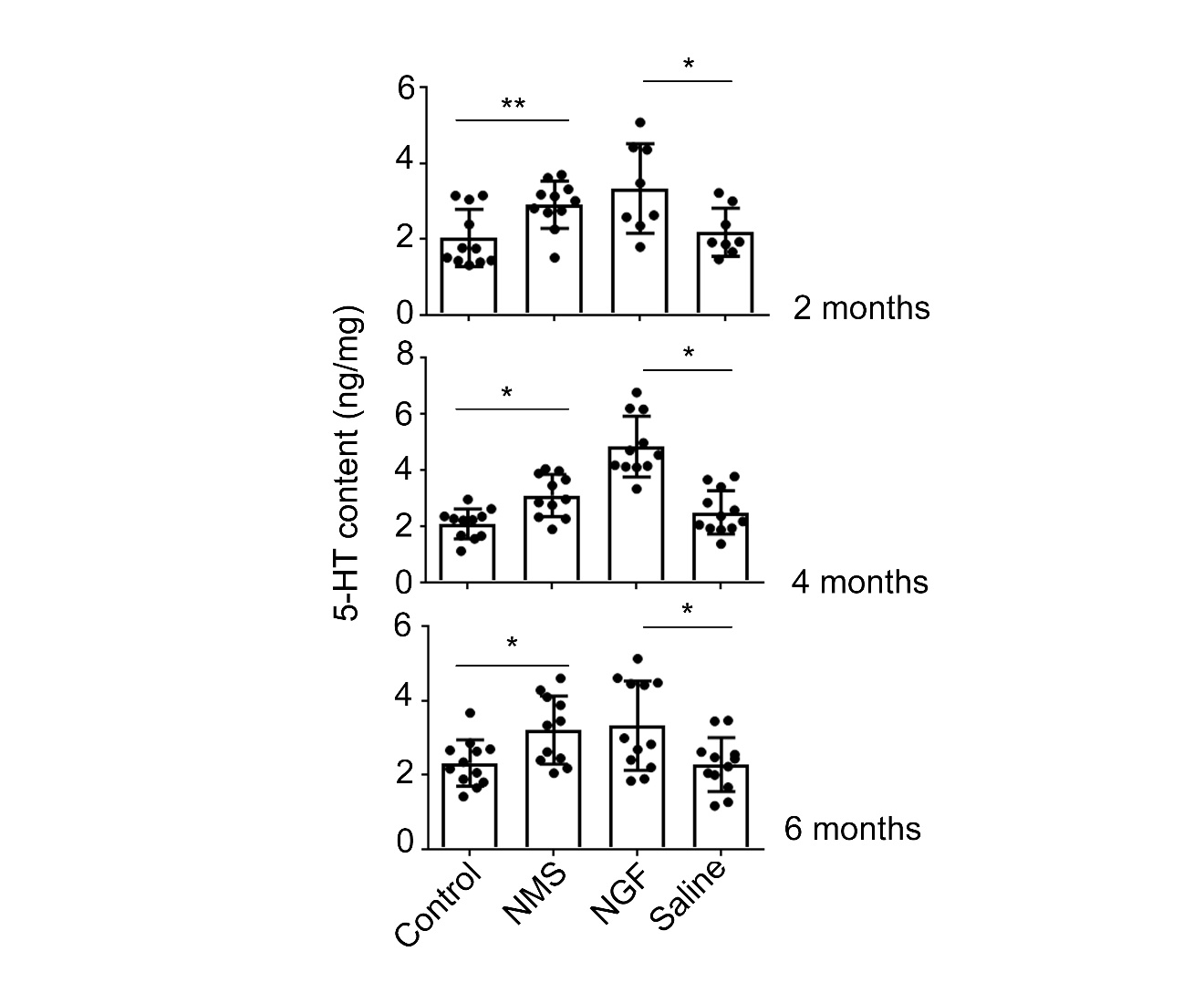
**

**Supplementary Figure 2 NMS results in increased production of colonic serotonin**

The concentration of serotonin/5HT in the proximal colons isolated from control and NMS mice treated with/without NGF was measured at indicated post-treatment times (2 months, 4 months, and 6 months) by capillary electrophoresis. (*p<0.05, **p<0.01; ***p<0.001, n≥8 /group; ANOVA)

**Supplementary Figure 3**

**
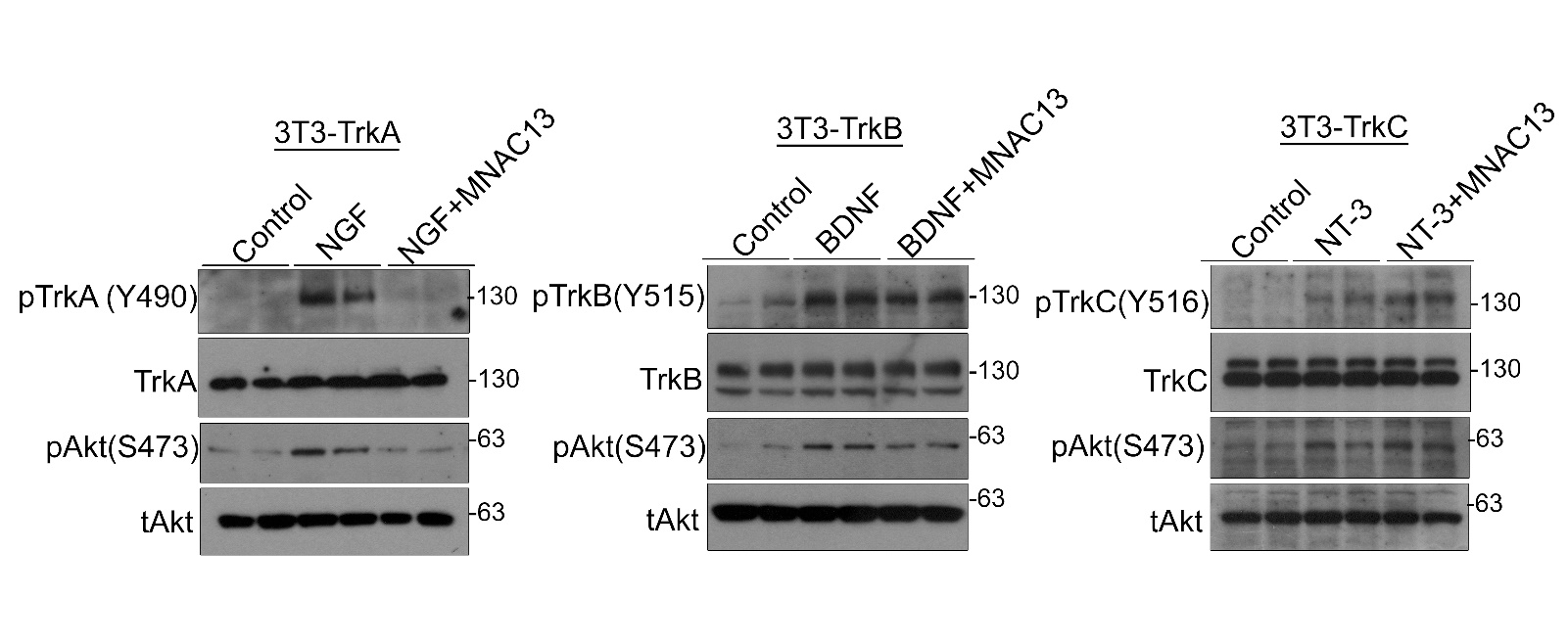
**

**Supplementary Figure 3. MNAC13 specifically inhibits TrkA activity and its downstream signaling**

Serum starved NIH-3T3 cells expressing TrkA, B or C were stimulated with either NGF or BDNF or NT-3 (20ng/ml) in the presence of indicated MNAC13 (100ng/ml). Representative western blots show the phosphorylation of Trks and Akt in response to the stimulation of various neurotrophic factors.

**Supplementary Figure 4**

**
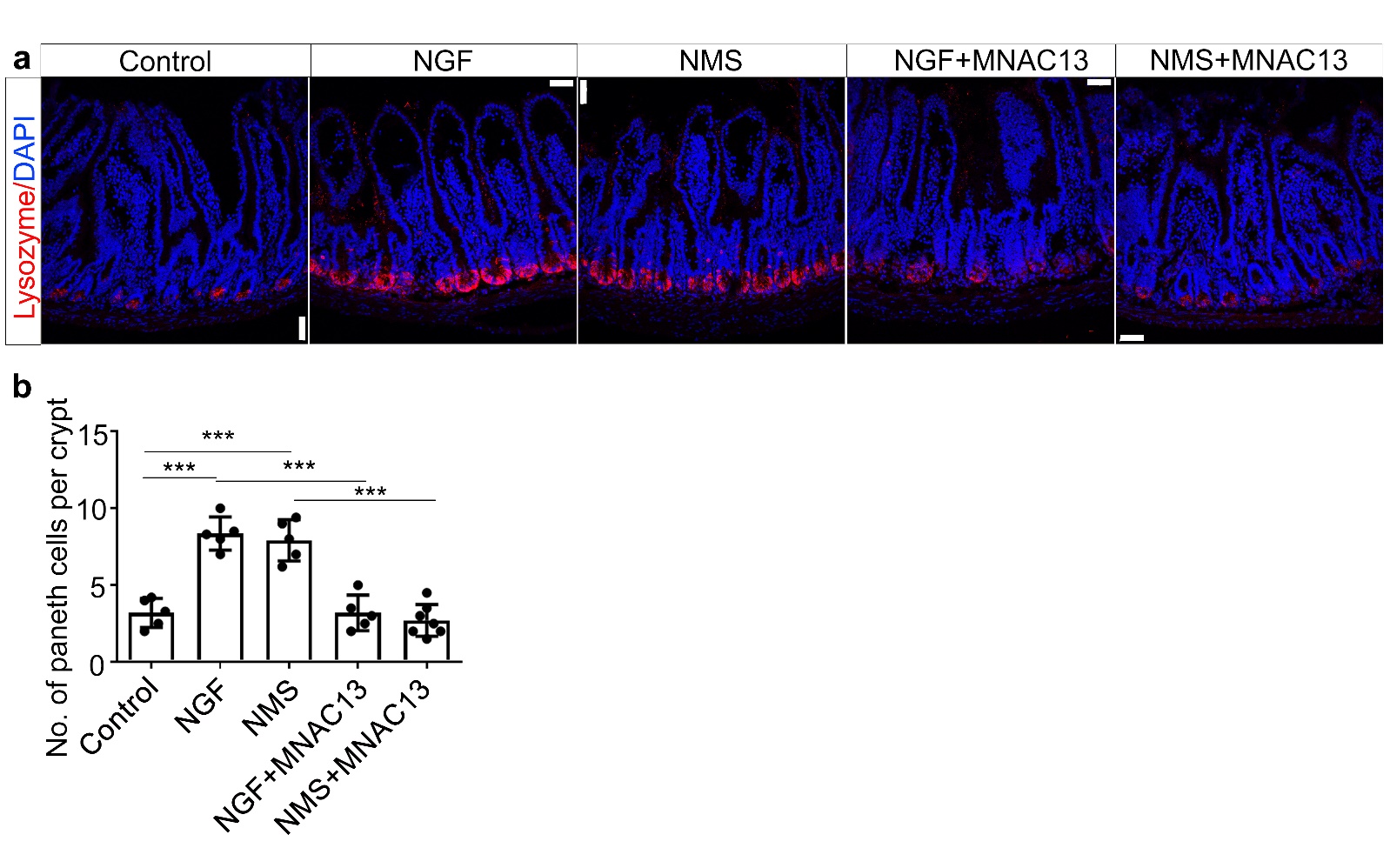
**

**Supplementary Figure 4. NMS expands the Paneth cell niche via NGF/TrkA signaling**

**(a)** Representative images showing Paneth cells in the small intestines from NMS and control mice treated with or without intraperitoneal injection of NGF and MNAC13. The Paneth cells were detected by immunostaining for lysozyme (red), a well-known marker for Paneth cells. (Scale bars: 50μm) Quantifications for their densities were shown in **(b)**. The density of Paneth cells was measured by the number of Paneth cells per crypt. (***p<0.001, n≥5/group, ANOVA).

**Supplementary Figure 5**

**
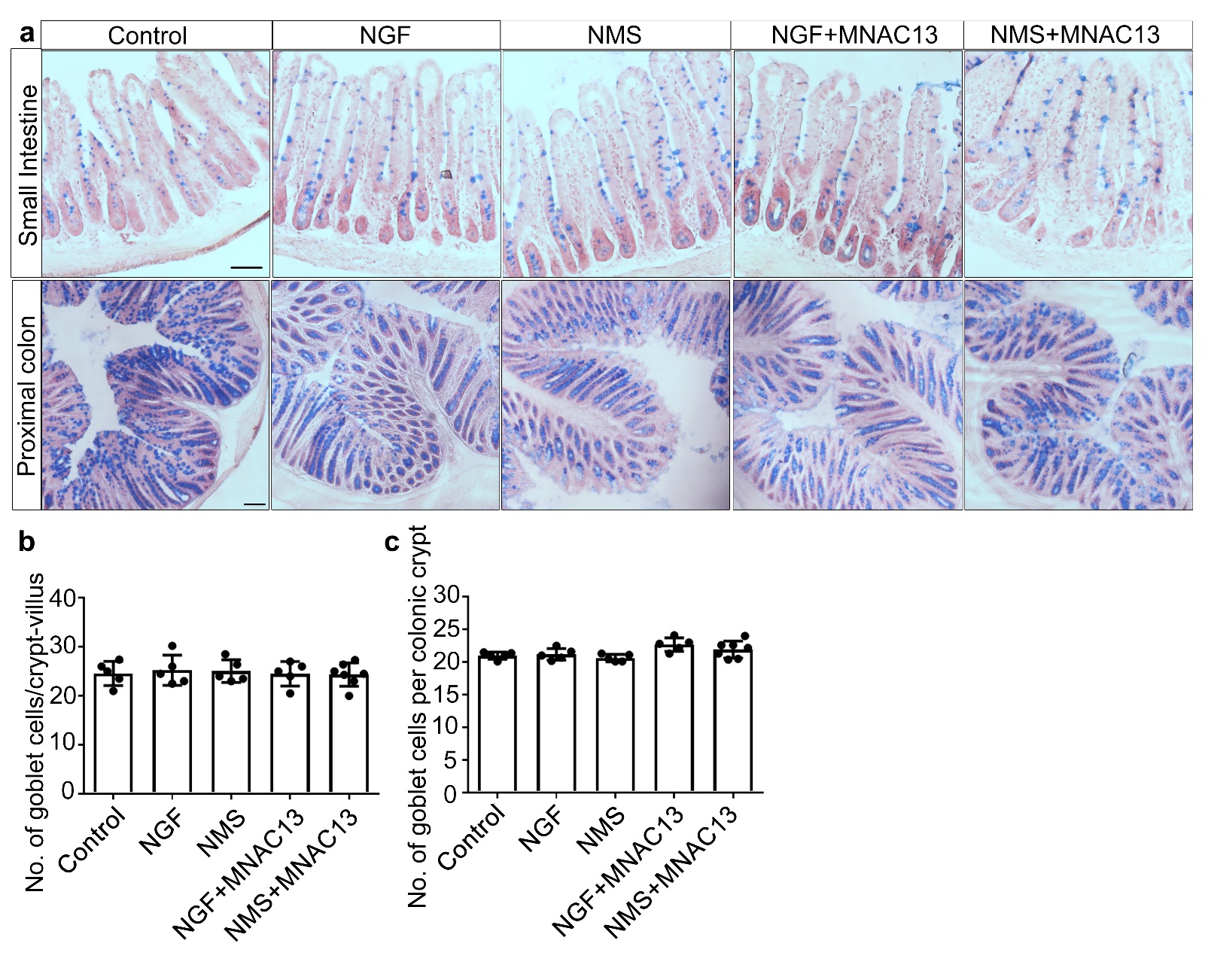
**

**Supplementary figure 5. NMS does not affect the Goblet cell niche in both small intestine and colon**

**(a**) Representative images showing and Goblet cells in both small intestines and proximal colons from NMS and control mice treated with or without intraperitoneal injection of NGF and MNAC13. The goblet cells were visualized by PAS staining. (scale bars: 100μm for small intestine images; 20μm for proximal colon images) Quantifications for the densities of goblet cell in colons and small intestines were shown in **(b)** and **(c)** respectively. The density of goblet cells was expressed as the number of PAS-positive cells per 10 crypt-villi. (***p<0.001, n≥5/group, ANOVA). Data represent the mean ± SEM**.**

**Supplementary Figure 6**

**
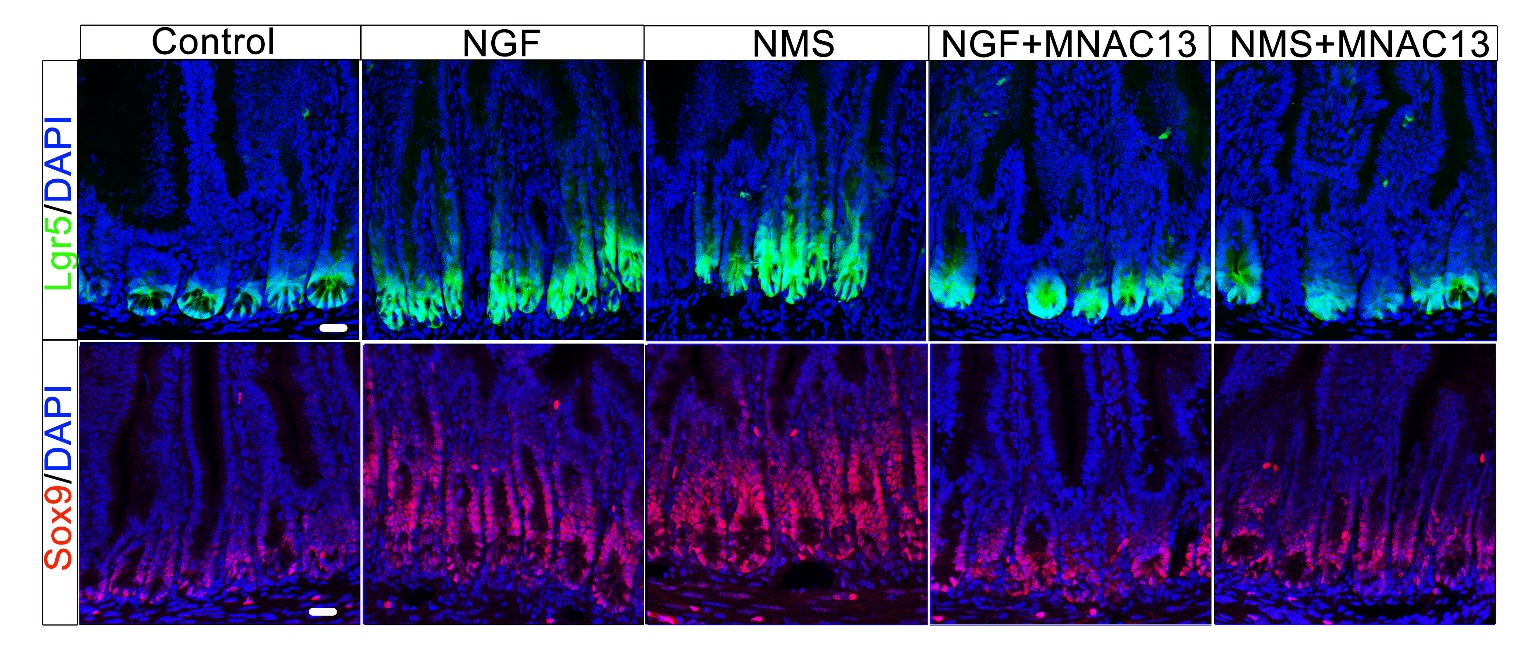
**

**Supplementary figure 6. NMS results in the expansion in intestinal stem cell compartment through NGF/TrkA signaling**

Intestinal stem cells were labelled with EGFP (green) and Sox9 (red) in the sections of small intestines from both control and NMS Lgr5-EGFP^+^ mice treated with or without intraperitoneal injection of NGF and MNAC13**.** Nuclear staining was visualized by DAPI (blue). (Scale bars: 20μm)

**Supplementary Figure 7**

**
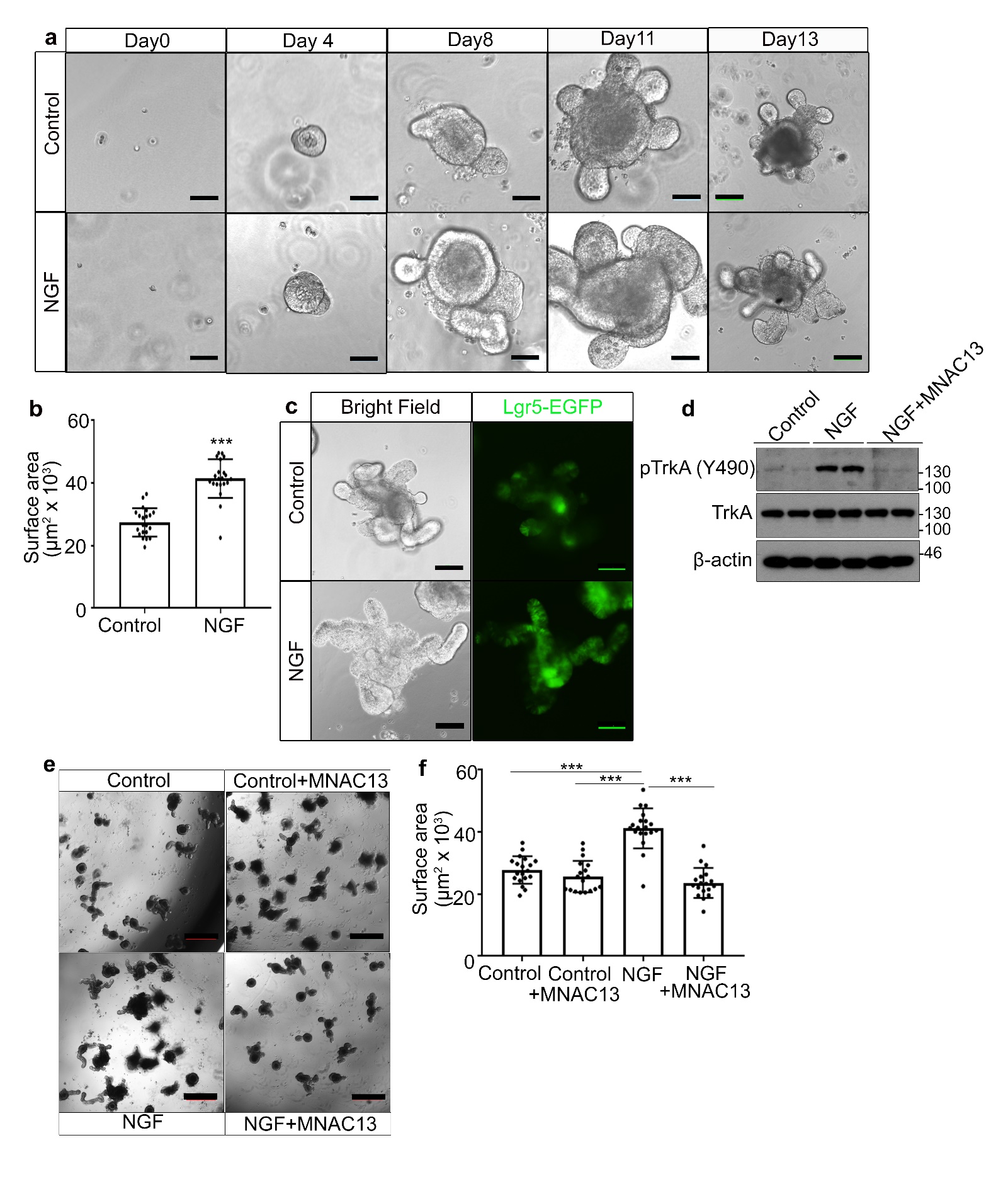
Supplementary figure 7. NGF enhances the functions of intestinal stem cells in organoid cultures**

**(a)** Lgr5-GFP^+^ ISCs were purified with FACS and cultured in matrigel with or without recombinant NGF. Representative images show the intestinal organoids cultured with or without recombinant NGF at various indicated time in culture. (Scale bars: 50μm for day 0-11, 100μm for day 13). (b) The size of organoids cultured with or without recombinant NGF (***p<0.001; n=20 organoids per group, two-tailed *t*-test) **(c)** Organoids treated with NGF maintain the Lgr5^+^ ISC marker. Phase contrast microscopy shows a typical organoid image from each model. The fluorescent images show that the majority of cells in the NGF-treated organoids remain Lgr5-GFP+ ISCs (green), while the control one is typical organoids with buds containing GFP+ ISCs at the tips (Right panel). (Scale bars: 100μm) **(d)** Western blotting analyses on the expression of phosphorylated TrkA (Y490) in the Lgr5-GFP^+^ ISC derived organoids that were treated with or without recombinant NGF and MNAC13 24h prior to the analyses. **(e)** Representative images show the intestinal organoids cultured with or without NGF and MNAC13 (Scale bars: 500μm). The size of organoids shown in **(e)** was quantified in **(f)** (***p<0.001; n=20 organoids per group, ANOVA) All data represent the mean ± SEM**.**

**Supplementary Figure 8**

**
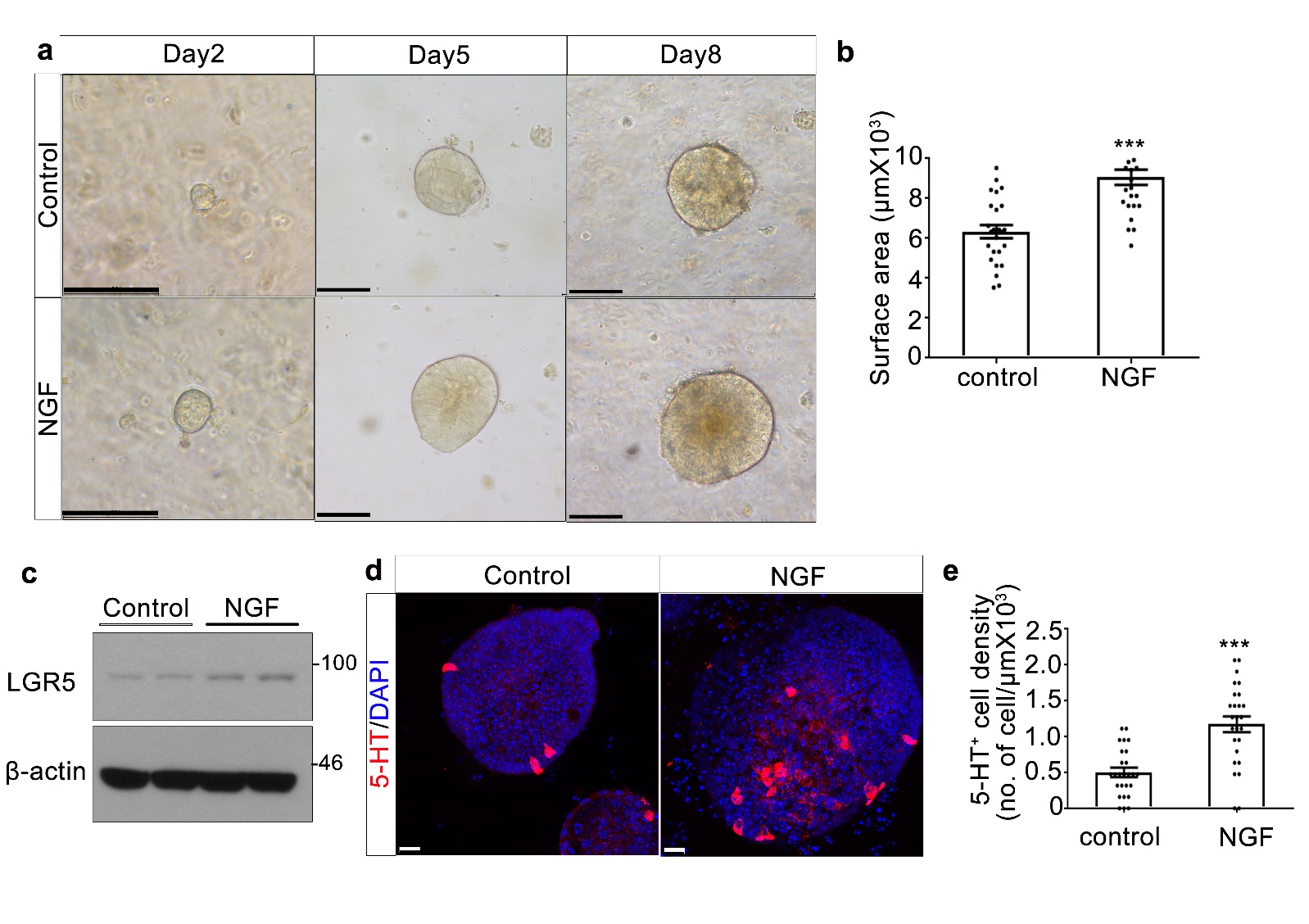
**

**Supplementary figure 8. NGF promotes the self-renewal of stem cells and EC cell generation in human colonic organoids**

**(a)** Representative images show the human colonic organoids cultured with or without recombinant human NGF (10ng/ml) at various indicated times in culture. (scale bar: 100μm) **(b)** The size of human organoids cultured with or without recombinant human NGF (***p<0.001; n=25 organoids per group, two-tailed *t*-test). **(c)** Western blotting analyses on the expression of LGR5 in the human organoids cultured with or without recombinant human NGF. (d) Confocal imaging shows whole mount-immunofluorescent co-staining for serotonin/5-HT (red) and DAPI (blue) in human colonic organoids cultured with/without NGF. (scale bar: 20 μm) Quantification for the density of 5-HT^+^ EC cells in organoids cultured with/without NGF was shown in **(e)**. (***p<0.001; n=25 organoids, two-tailed *t*-test) All data represent the mean ± SEM**.**

**Supplementary Figure 9**

**
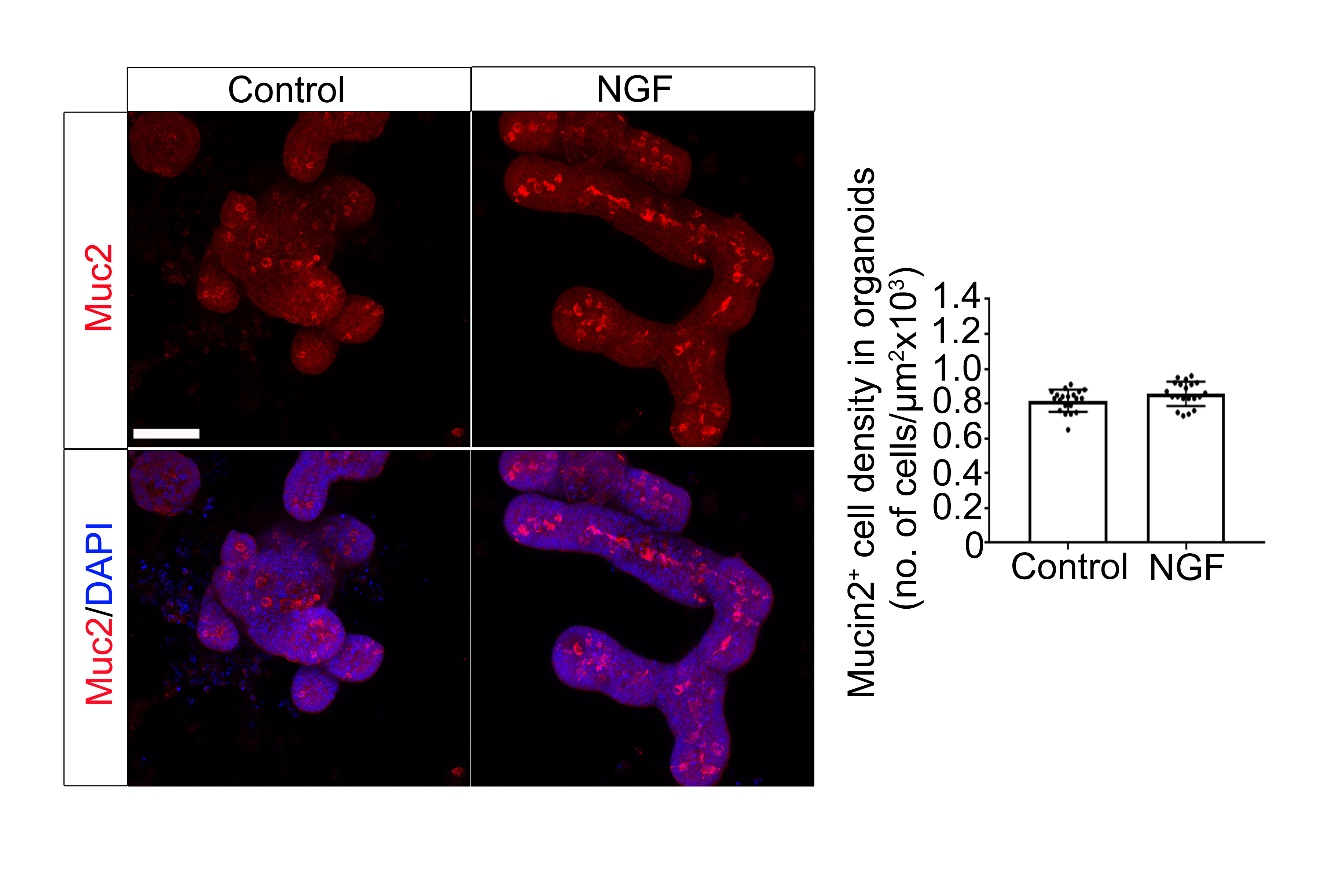
**

**Supplementary Figure 9. NGF does not alter the differentiation of goblet cells in the intestinal organoid**

Wholemount co-staining for Mucin-2 (Muc2) (red) and DAPI (blue) in intestinal organoids cultured with/without NGF (left panel)**.** Quantification for the density of Muc2^+^ goblet cells was shown in the right panel. Data represent the mean ± SEM**.** (Scale bars: 50μm)

**Supplementary Figure 10**

**
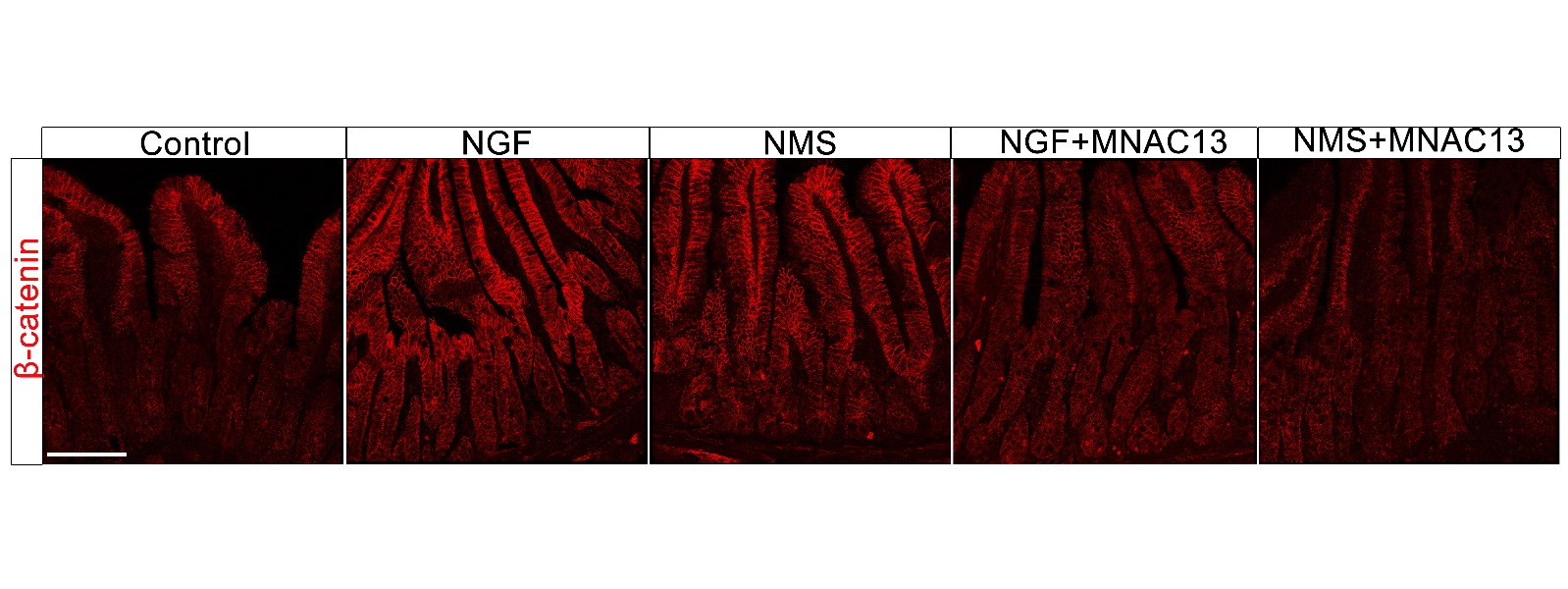
**

**Supplementary Figure 10. NMS, mediated via NGF/TrkA signaling, increased β-catenin expression in mice**

Immunofluorescent staining for β-catenin (red) in the sections of small intestines from control and NMS mice treated with or without intraperitoneal injection of NGF and MNAC13. Nuclei were counterstained with DAPI. (Scale bars: 50μm)

**Supplementary Figure 11**

**
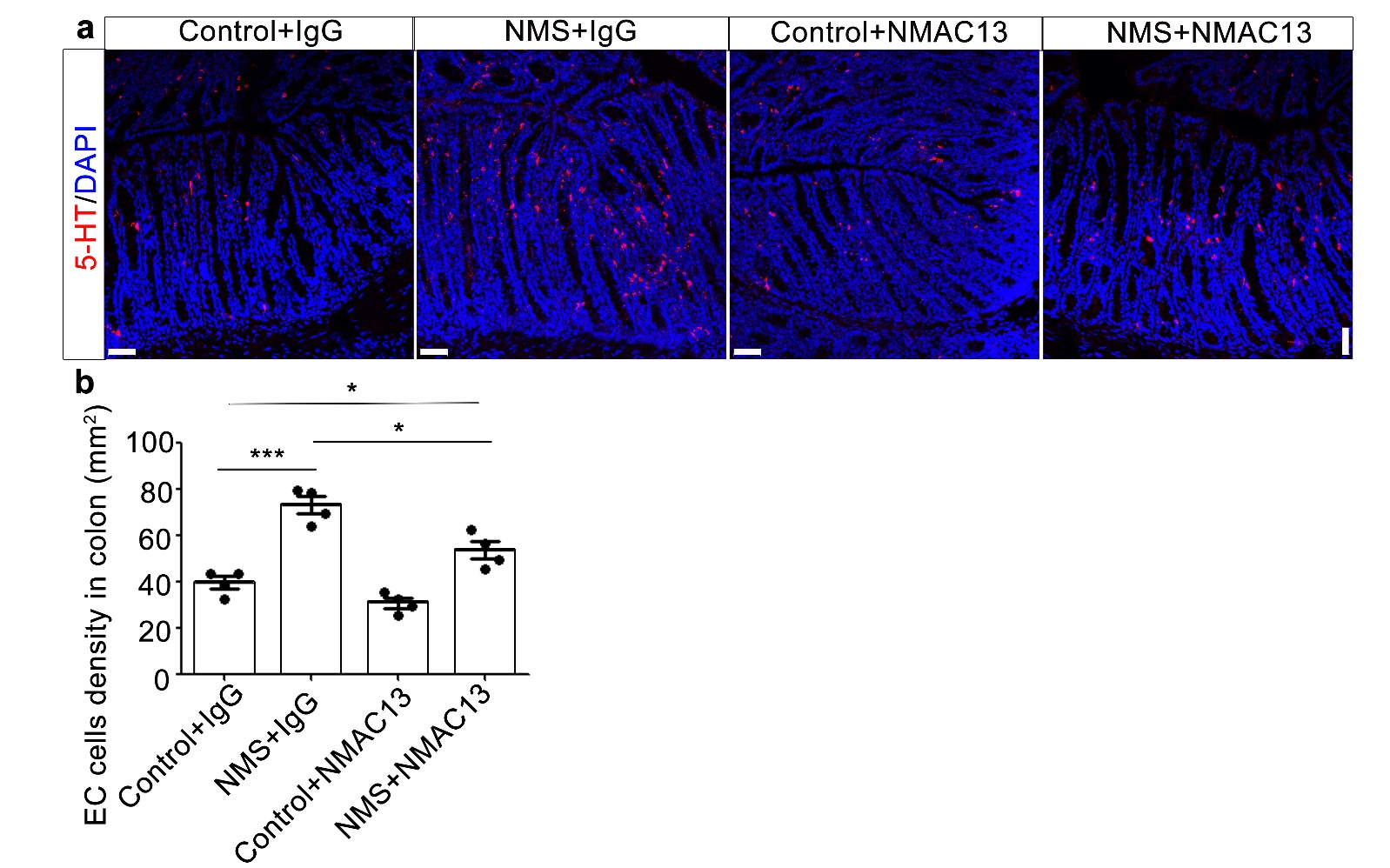
**

**Supplementary Figure 11. Inhibiting TrkA rescues the EC cell hyperplasia in NMS mice in the adult life**

**(a)** Representative images showing serotonin-producing EC cells (red) in in the proximal colons from NMS and control mice treated with or without intraperitoneal injection of NMAC13. NMS mice at the age of 10 weeks were daily intraperitoneally injected with either NMAC13 or control IgG for 10 days. Nuclei were counterstained with DAPI. (Scale bars: 50μm) Quantification for EC cell density (the number of EC cells per mm^2^ of colonic mucosa) and the number of EGFP^+^ cells per colonic crypts were shown **(b)**. (*p<0.05, ***p<0.001; n=4/group; ANOVA)

**Supplementary Figure 12**

**
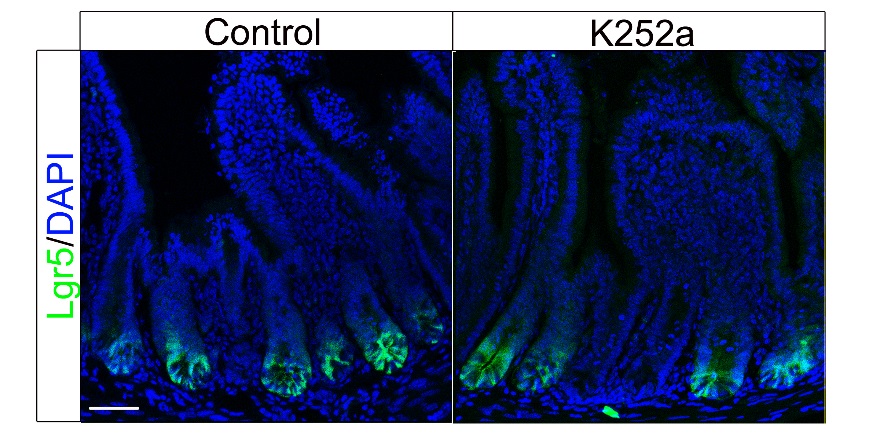
**

**Supplementary Figure 12. Inhibition of NGF/TrkA signaling does not affect intestinal stem cell renewal**

Confocal imaging shows the distribution of Lgr5^+^ ISCs (green) in small intestines isolated from Lgr5-EGFP^+^ mice treated with/without daily intraperitoneal injection of K252a during postnatal stage from p3 to p14. Nuclei were stained with DAPI (blue). (Scale bars: 50μm)

**Supplementary Figure 13**

**
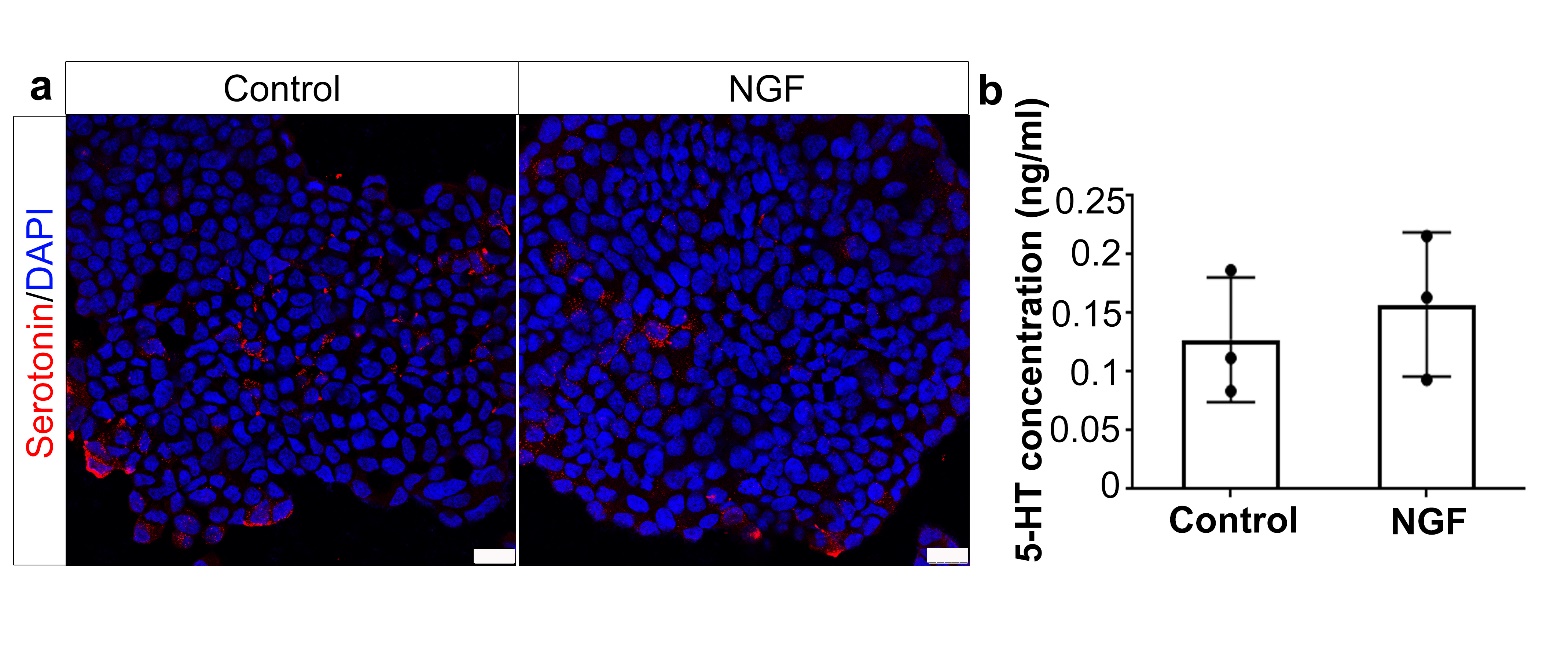
**

**Supplementary Figure 13. Exogenous NGF does not affect the biosynthesis of serotonin in neuroendocrine cells**

**(a-b)** Serum-starved QGP cells were treated with or without recombinant NGF for 24 hours. **(a)** The production of serotonin in QGP cells was then visualized by immunostaining for serotonin (red). Nuclei were labelled with DAPI (blue). (Scale bars: 25μm). **(b)** The content of 5-HT in the conditioned media of QGP cells was measured by ELISA. (n=3) Data represent the mean ± SEM**.**

**Supplementary Figure 14**


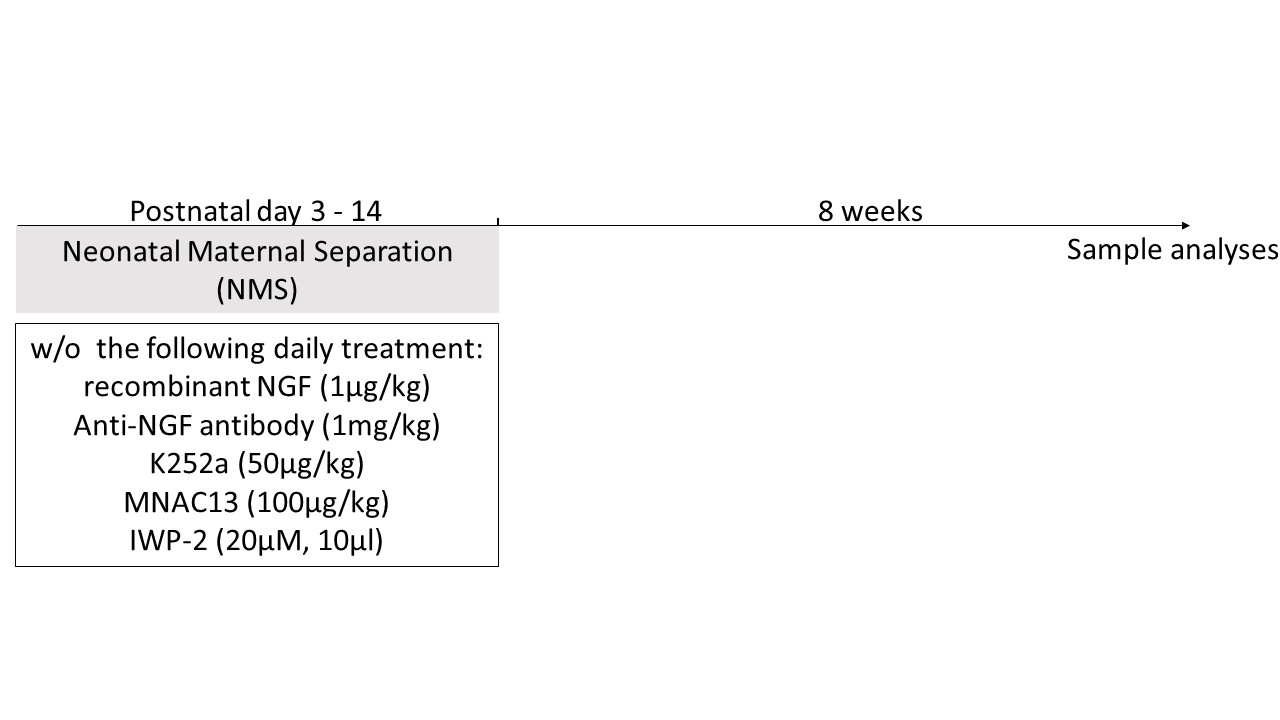


**Supplementary Figure 14. Timeframe for drug treatments**

A diagram shows the experimental design for various drug treatment in this study.

**Supplementary Figure 15**


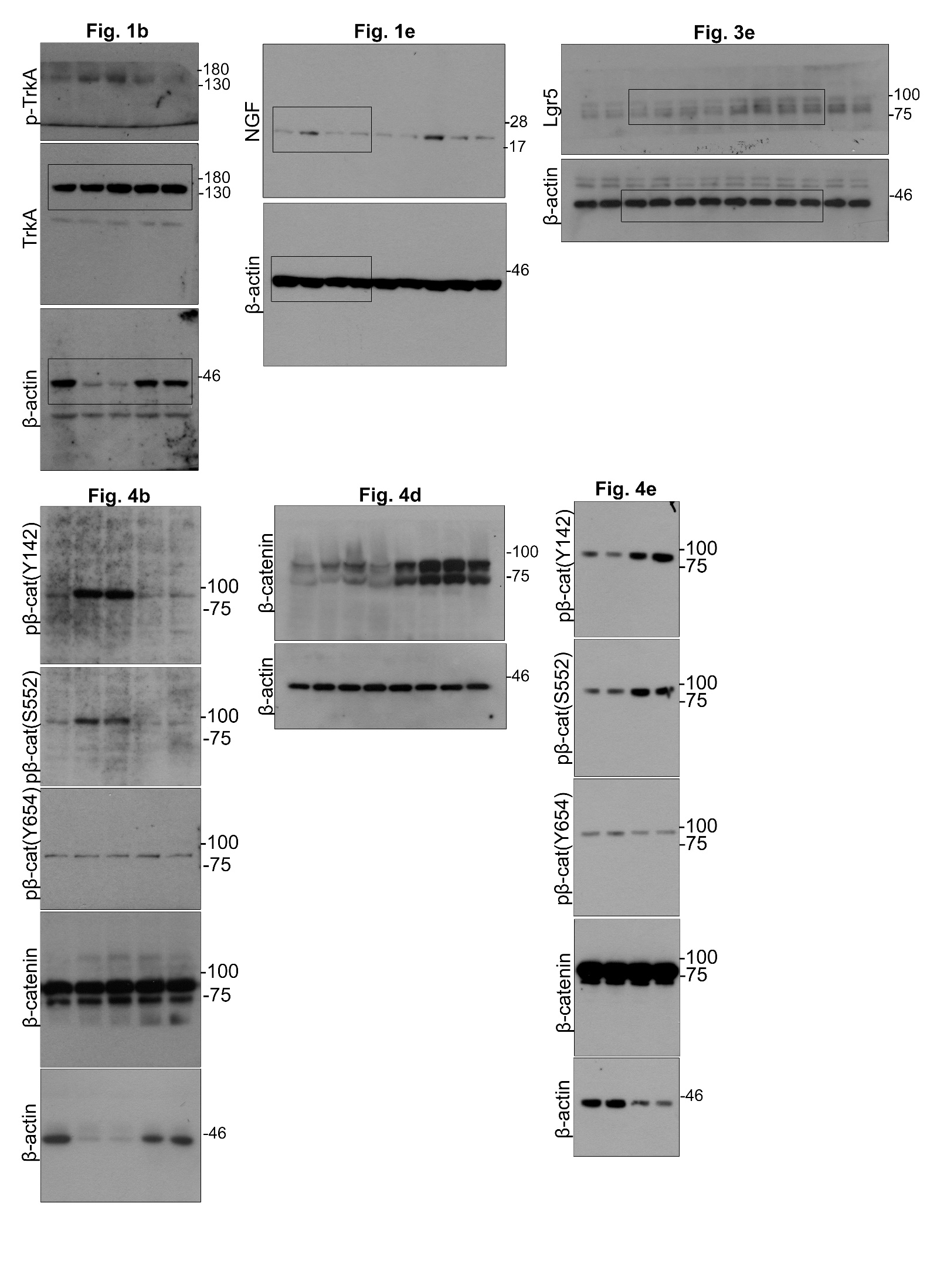


**Supplementary Figure 15 The original immunoblots for all main figures and supplementary figures.** Black bloxed area represents where a panel in each immunoblot was selected for display.

**Supplementary Figure 15 (Continued)**


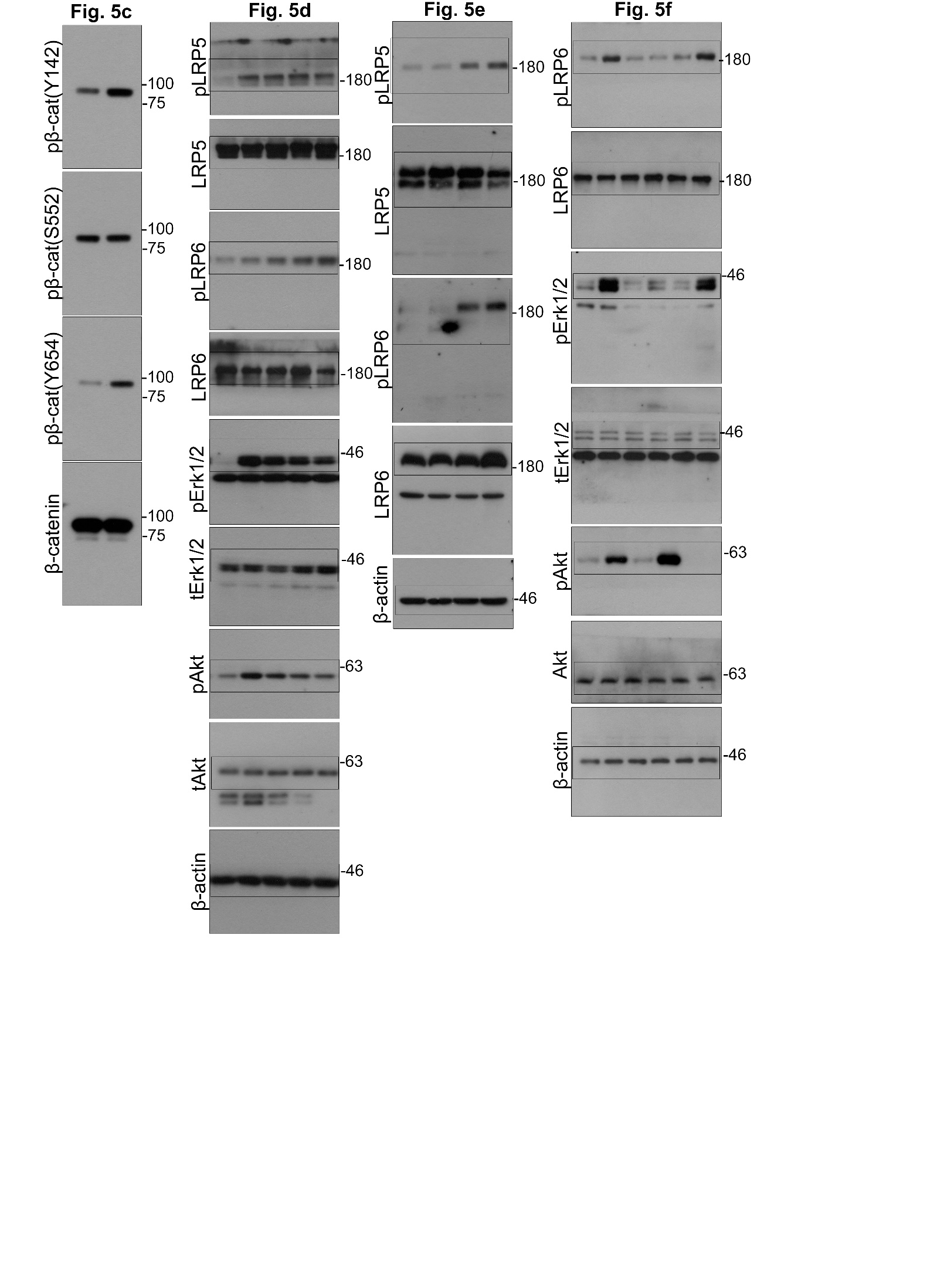


**Supplementary Figure 15 (Continued)**


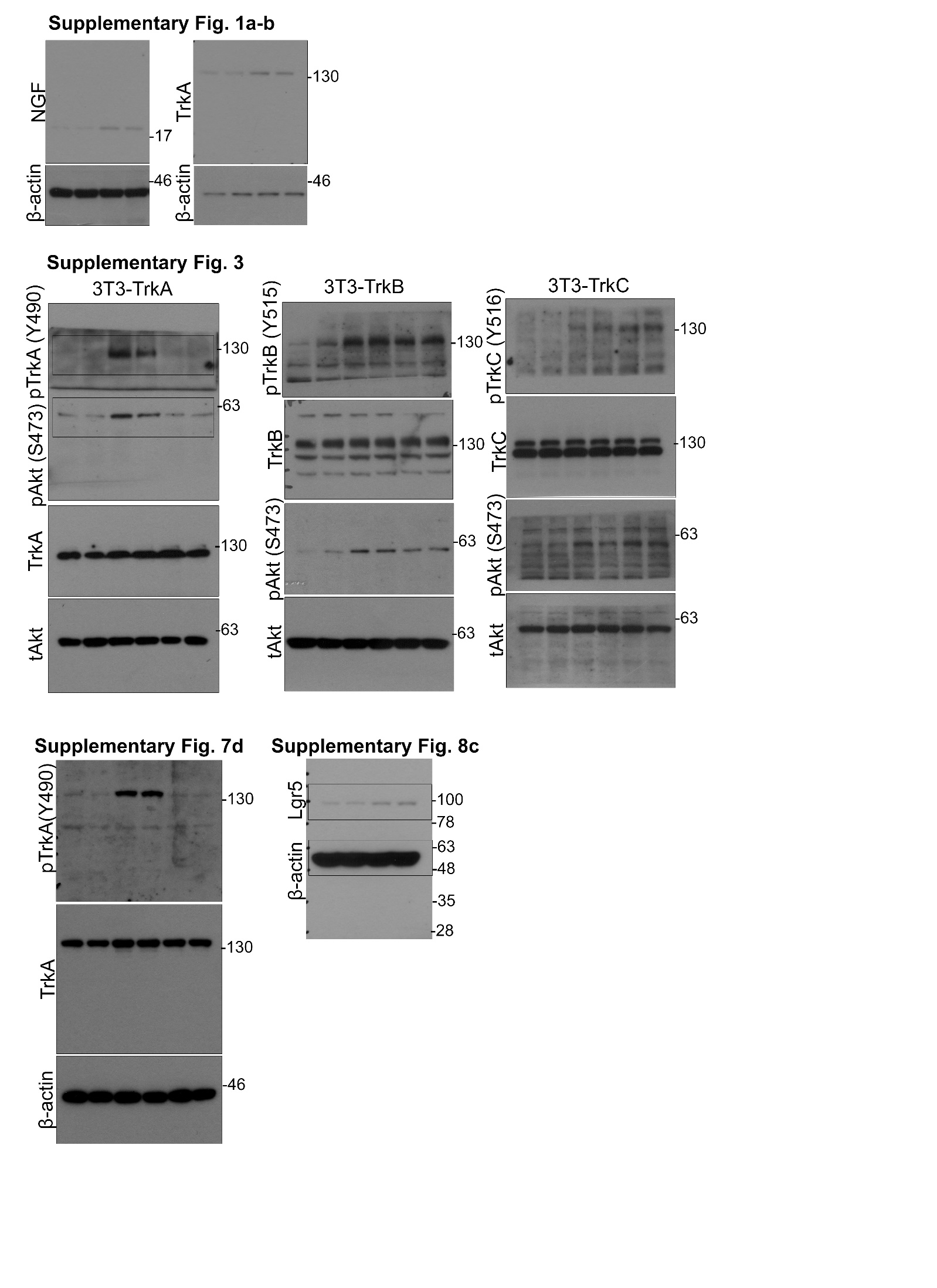


**Supplementary Table 1**

| **Target** | **Sense (5’-3’)** | **Antisense (5’-3’)** |
| --- | --- | --- |
| *Lgr5* | CGAGCCTTACAGAGCCTGATACC | TTGCCGTCGTCTTTATTCCATTGG |
| *Sox9* | CTGGAGGCTGCTGAACGAGAG | CGGCGGACCCTGAGATTGC |
| *β-catenin* | TGACACCTCCCAAGTCCTTT | TTGCATACTGCCCGTCAAT |
| *Axin2* | GAGAGTGAGCGGCAGAGC | CGGCTGACTCGTTCTCCT |
| *Cd44* | TCGATTTGAATGTAACCTGCCG | CAGTCCGGGAGATACTGTAGC |
| *Ascl2* | CCTCTCTCGGACCCTCTCTCAG | CAGTCAAGGTGTGCTTCCATGC |
| *Gapdh* | AGCTTGTCATCAACGGGAAG | TTTGATGTTAGTGGGGTCTCG |

**List of primers used in the study**
