# Supplementary material for: Early life stress disrupts intestinal homeostasis via NGF-TrkA signaling
Source: Nat Commun. 2019 Apr 15;10:1745. doi: 10.1038/s41467-019-09744-3 (PMC6465335; doi:10.1038/s41467-019-09744-3)
Supplement: Supplementary file 3 — Reporting Summary [file 41467_2019_9744_MOESM3_ESM.pdf]

nature research

Corresponding author(s): Bian Zhaoxiang

Last updated by author(s): Jan 29, 2019

Reporting Summary

Nature Research wishes to improve the reproducibility of the work that we publish. This form provides structure for consistency and transparency in reporting. For further information on Nature Research policies, see Authors & Referees and the Editorial Policy Checklist.

Statistics

For all statistical analyses, confirm that the following items are present in the figure legend, table legend, main text, or Methods section.

n/a	Confirmed	
☐	☒	The exact sample size (*n*) for each experimental group/condition, given as a discrete number and unit of measurement
☐	☒	A statement on whether measurements were taken from distinct samples or whether the same sample was measured repeatedly
☐	☒	The statistical test(s) used AND whether they are one- or two-sided<br>*Only common tests should be described solely by name; describe more complex techniques in the Methods section.*
☐	☒	A description of all covariates tested
☐	☒	A description of any assumptions or corrections, such as tests of normality and adjustment for multiple comparisons
☐	☒	A full description of the statistical parameters including central tendency (e.g. means) or other basic estimates (e.g. regression coefficient) AND variation (e.g. standard deviation) or associated estimates of uncertainty (e.g. confidence intervals)
☐	☒	For null hypothesis testing, the test statistic (e.g. *F*, *t*, *r*) with confidence intervals, effect sizes, degrees of freedom and *P* value noted<br>*Give P values as exact values whenever suitable.*
☒	☐	For Bayesian analysis, information on the choice of priors and Markov chain Monte Carlo settings
☒	☐	For hierarchical and complex designs, identification of the appropriate level for tests and full reporting of outcomes
☐	☒	Estimates of effect sizes (e.g. Cohen's *d*, Pearson's *r*), indicating how they were calculated

Our web collection on statistics for biologists contains articles on many of the points above.

Software and code

Policy information about availability of computer code

Data collection	LAS X by Leica was used for confocal imaging.
Data analysis	Prism 7 and Microsoft Excel were used to generate graphs and to perform the statistical analyses. The quantification of protein expression in western blots were analyzed by Image J.

For manuscripts utilizing custom algorithms or software that are central to the research but not yet described in published literature, software must be made available to editors/reviewers. We strongly encourage code deposition in a community repository (e.g. GitHub). See the Nature Research guidelines for submitting code & software for further information.

Data

Policy information about availability of data

All manuscripts must include a data availability statement. This statement should provide the following information, where applicable:

- Accession codes, unique identifiers, or web links for publicly available datasets
- A list of figures that have associated raw data
- A description of any restrictions on data availability

The data that support the findings of this study are available from the corresponding author upon reasonable request.

Field-specific reporting

Please select the one below that is the best fit for your research. If you are not sure, read the appropriate sections before making your selection.

☒ Life sciences ☐ Behavioural & social sciences ☐ Ecological, evolutionary & environmental sciences

October 2018

Life sciences study design

All studies must disclose on these points even when the disclosure is negative.

Sample size	Sample size were chosen to generate reproducible results with desirable significance (0.05) and power (>90%).
Data exclusions	No data was excluded from the analyses.
Replication	All of the experimental results were replicated as indicated in figure legends. For in vitro experiments, each experiment was independently repeated at least three times. Only biological replicates were plotted and used for statistical analyses.
Randomization	Tissues from at least three independent and randomly chosen mice at comparable developmental stages were collected for analyses and none
Blinding	Investigators were not blinded to group allocation in data collection and analyses.

Reporting for specific materials, systems and methods

We require information from authors about some types of materials, experimental systems and methods used in many studies. Here, indicate whether each material, system or method listed is relevant to your study. If you are not sure if a list item applies to your research, read the appropriate section before selecting a response.

Materials & experimental systems

n/a	Involved in the study	
☐	☒	Antibodies
☐	☒	Eukaryotic cell lines
☒	☐	Palaeontology
☐	☒	Animals and other organisms
☐	☒	Human research participants
☒	☐	Clinical data

Methods

n/a	Involved in the study	
☒	☐	ChIP-seq
☒	☐	Flow cytometry
☒	☐	MRI-based neuroimaging

Antibodies

Antibodies used	The antibodies used in this study include the following: anti-TrkA (sc-118, Santa Cruz); anti-phospho-TrkA (Tyr490) (PA5-17877, Thermo Fisher) anti-NGF (N6655, Sigma); anti-lysozyme (Rb-372-R7, Thermo Scientific); anti-Sox9 (AB5535, Millipore); anti-Chromogranin A (sc-13090, Santa Cruz); anti-Mucin2 (sc-15334, Santa Cruz); anti-Serotonin (ab66047, abcam); anti-β-catenin (8480, Cell signaling); anti-p-β-catenin (Tyr-142)(CP10811, ECM Biosciences); anti-p-β-catenin (S552)(9566, Cell signaling,); anti-p-β-catenin (Tyr-654)(sc-57533, Santa Cruz); anti-Akt (4685, Cell signaling): anti-pAkt (4060, Cell signaling); anti-Erk1/2 (4695, Cell signaling,); anti-pErk1/2 (9101, Cell signaling); anti-LRP5 (5731, Cell signaling); anti-pLRP5 (ab203306, Abcam); anti-LRP6 (2560, Cell signaling); anti-pLRP6 (2568, Cell signaling); anti-β-actin (12262, Cell signaling); Alexa Fluor 594-conjugated goat anti-rabbit (A-11012, Invitrogen); Alexa Fluor 594-conjugated donkey anti-goat antibody (A11058, Invitrogen); Goat anti-rabbit antibody conjugated with HRP (sc-2030, Santa Cruz,); Goat anti-mouse antibody conjugated with HRP (sc-2031, Santa Cruz)
Validation	Validations are based on the datasheets from the manufacturers of antibodies.

Eukaryotic cell lines

Policy information about cell lines

Cell line source(s)	HEK293T cells and Caco2 cells were provided by Professor Zhou Zhongjun at the University of Hong Kong.
Authentication	None of the cell lines were authenticated.
Mycoplasma contamination	All cell lines are negative for mycoplasma.
Commonly misidentified lines (See ICLAC register)	No commonly misidentified cell lines were used.

Animals and other organisms

Policy information about studies involving animals; ARRIVE guidelines recommended for reporting animal research

Laboratory animals	Sprague-Dawley (SD) rats were obtained from the Laboratory Animal Services Centre of The Chinese University of Hong Kong,

Laboratory animals	Hong Kong SAR, China. Lgf5-EGFP-IRES-CreET2 mice on C57BL/6J background were obtained from the Jackson laboratory. All animals and their borne pups were housed in the animal room at Hong Kong Baptist University, kept on a 12-hour (h) light/dark cycle with constant ambient temperature, fed with standard laboratory chow and applied with water ad libitum. Animals of both sexes were used in experiments. Exact numbers of animals used in individual experiments are indicated in the figure legends.
Wild animals	No wild animals was used.
Field-collected samples	This study did not involve samples collected from the fields.
Ethics oversight	All animal experiments were performed in accordance to the guideline of the Committee on the Use of Human & Animal Subjects in Teaching & Research at Hong Kong Baptist University.

Note that full information on the approval of the study protocol must also be provided in the manuscript.

Human research participants

Policy information about studies involving human research participants

Population characteristics	IBS patients were diagnosed according to Rome III criteria. 45 IBS patients and 31 healthy controls with mean age of 49 years were recruited for the study. As serotonin level is usually associated with IBS with diarrhea predominance, we included mainly IBS patients with diarrhea (IBS-D) for the experiment.<br>Patients will be included if they have all the follows: 1) meet of diagnostic criteria for IBS (Rome III); 2) age of 18 to 65 years; 3) IBS symptom severity Scale (IBS-SSS) > 75 points at baseline and during the 2-week run-in period; 4) Normal colonic evaluation (colonoscopy or barium enema) within 5 years.<br>Patients will be excluded if they have one or more of follows: 1) pregnancy or breast-feeding; 2) medical history of inflammatory bowel diseases, carbohydrate malabsorption, hormonal disorder, known allergies to food additives, and/or any other serious diseases; 3) having suicidal intention or attempts or aggressive behavior; 4) Use of medications known to influence gastrointestinal transit.<br>Blood samples were collected from healthy subjects and IBS patients in the morning by fasting for 12 hours.
Recruitment	Informed consent was obtained from each patient and healthy control. The participants were recruited from clinics of School of Chinese Medicine at Hong Kong Baptist University.
Ethics oversight	The study protocol was approved by the Committee on the Use of Human & Animal Subjects in Teaching & Research at Hong Kong Baptist University.

Note that full information on the approval of the study protocol must also be provided in the manuscript.
